# *Notes from the Field:* Multidrug-Resistant Tuberculosis Among Workers at Two Food Processing Facilities — Ohio, 2018–2019

**DOI:** 10.15585/mmwr.mm6932a6

**Published:** 2020-08-14

**Authors:** Amish Talwar, Rebekah Stewart, Sandy P. Althomsons, Jessica Rinsky, David A. Jackson, Maria E. Galvis, Philip Graham, Moises A. Huaman, James Karrer, Karthik Kondapally, Sarah Mitchell, Jonathan Wortham, Sietske de Fijter

**Affiliations:** ^1^Division of Tuberculosis Elimination, National Center for HIV/AIDS, Viral Hepatitis, STD, and TB Prevention, CDC; ^2^Epidemic Intelligence Service, CDC; ^3^National Institute for Occupational Safety and Health, CDC; ^4^Hamilton County Public Health, Ohio; ^5^Butler County General Health District, Ohio; ^6^Ohio Department of Health.

During 2018–2019, the Ohio Department of Health (ODH) reported three cases of multidrug-resistant tuberculosis (MDR TB)* in persons who worked in two food processing facilities. The National Tuberculosis Molecular Surveillance Center^†^ performed whole genome sequencing of a *Mycobacterium tuberculosis* isolate from each patient; phylogenetic analysis revealed the isolates were genetically identical. Prompted by concern for MDR TB transmission associated with these workplaces and surrounding communities, ODH began an investigation in February 2019. CDC was invited to assist with the investigation and deployed a team to Ohio on April 14, 2019.

The CDC-ODH team, which included representatives from CDC’s Division of Tuberculosis Elimination and the National Institute for Occupational Safety and Health (NIOSH), reviewed medical and employment records, conducted principal informant interviews, and conducted a tour of one of the facilities (facility A) where the three patients worked. The third patient also worked at a second facility (facility B), which had closed as part of an unrelated business restructuring before the CDC-ODH team could begin its investigation; facility A remained operational throughout the investigation. A separate NIOSH team had visited facility B before it closed to conduct a health hazard evaluation following notification that one the facility’s employees had MDR TB; observations from that visit were used to guide the exposure assessment of facility B employees. The index case occurred in a person born in one of the 30 countries designated by the World Health Organization as having a high prevalence of MDR TB (*1*). According to available work schedules, during the index patient’s infectious period, the second and third patients had worked for at least 54 days and 7 days,^§^ respectively, on the same food production line as the index patient. The investigation team was unable to find any other potential transmission venues or common exposures among the three patients.

No additional cases of MDR TB related to this group of patients were identified. However, 971 contacts of the three MDR TB patients were identified, including 941 who were workplace contacts; the majority of contacts were non–U.S.-born persons. Contacts were prioritized according to levels of possible TB exposure; 478 contacts, including 448 workplace and 30 personal contacts, had the highest risk of exposure (high-priority contacts).^¶^ As of April 26, 2019, a total of 160 (36%) of the 448 high-priority workplace contacts had been tested for TB infection, 59 (37%) of whom had positive results for a tuberculin skin test or interferon-γ release assay test, both of which test for TB infection. Among those with positive test results, 19 (32%) began latent tuberculosis infection treatment (Table). Among the overall U.S. population, an estimated 21% of non–U.S.-born persons have a positive tuberculin skin test in the United States, and 16% have a positive interferon-γ release assay result (*2*). The higher percentage of positive TB test results at the workplace provides evidence for likely workplace transmission. Based on principal informant interviews, likely contributors to the low level of TB testing and treatment for infection among contacts included difficulties in communication, perceived barriers to care, and mistrust of government authorities.

**TABLE:** Tuberculosis (TB) care cascade for high-priority* contacts of three patients with multidrug-resistant TB — Ohio, April 2019

Contact type	No. of high-priority contacts	No. (%)		
		Tested^†^	Tested, with positive TB test result^†^	Tested, with positive TB test result and started on LTBI treatment
**Workplace**	448	160 (36)	59 (37)	19 (32)
Facility A	247	120 (49)	39 (33)	19 (49)
Facility B	201	40 (20)	20 (50)	0^§^ (0)
**Personal^¶^**	30	16 (53)	13 (81)	8 (62)
**Total**	**478**	**176 (37)**	**72 (41)**	**27 (38)**

**Abbreviation:** LTBI = latent tuberculosis infection.

* Includes named contacts and workplace contacts with documented direct exposure to a multidrug-resistant (MDR) TB patient, health care workers with documented direct exposure to an MDR TB patient when the patient was contagious and not under airborne infection isolation, and contacts with risk factors for TB, such as human immunodeficiency virus infection, diabetes mellitus, end stage renal disease, or immunosuppression.

^†^ Includes five contacts who were tested with interferon-γ release assay (QuantiFERON-TB Gold In-Tube test), three of whom had positive test results (all personal contacts).

^§^ Initiation of treatment was pending drug-susceptibility testing results as of April 26, 2019.

^¶^ Includes contacts who spent substantial time with patients at home.

After the investigation concluded on April 26, 2019, all three patients with MDR TB disease had either recovered or were continuing to recover, and no additional cases have been identified. ODH continues to work with its local partners to facilitate TB testing and treatment of contacts with latent TB infection and to monitor for new cases.

MDR TB is rare in the United States (<3% of TB cases annually since 1993) (*3*,*4*); in 2018, there were 98 MDR TB cases in the United States out of a total of 9,025 TB cases (*5*). Although the TB transmission source for the index patient remains uncertain, the low prevalence of MDR TB in the United States and the absence of other genotype-matched TB cases in the national TB molecular surveillance database indicate that the patient was likely infected in the patient’s country of origin. Given the nonspecific signs and symptoms of TB, health care providers should consider TB when examining persons with cough, chest pain, hemoptysis, weight loss, fever, chills, night sweats, weakness, fatigue, or loss of appetite, especially when the person has TB risk factors, including birth in areas with high rates of TB.**^,^^††^ In addition, providers should consider prompt molecular detection of drug-resistance testing for TB patients with risk factors for drug-resistant TB.^§§^^,^^¶¶^ Finally, public health agencies need to facilitate engagement with communities with higher rates of TB to build trust, which is important for successful disease investigations. Activities might include communicating in a culturally sensitive manner with community members, offering patients incentives for getting tested or treated, providing transportation to clinics, using mobile clinics, and conducting communitywide education efforts.

## Footnotes

* MDR TB is a form of tuberculosis caused by *M. tuberculosis* resistant to isoniazid and rifampin, two cornerstone drugs used in the first-line TB treatment regimen.

§ These reflect minimum counts because complete daily employment records were unavailable.

¶ High-priority contacts include named contacts and workplace contacts with documented direct exposure to an MDR TB patient, health care workers with documented direct exposure to an MDR TB patient when the patient was contagious and not under airborne infection isolation, and contacts with risk factors for TB, such as human immunodeficiency virus infection, diabetes mellitus, end stage renal disease, or immunosuppression.

** https://www.cdc.gov/tb/topic/basics/signsandsymptoms.htm.

†† https://www.cdc.gov/tb/topic/basics/risk.htm.

§§ Risk factors for drug-resistant TB include failure to adhere to or complete TB treatment, incorrect TB treatment (i.e., incorrect dose or length of treatment prescribed), prior TB treatment, residence in areas of the world where drug-resistant TB is common, and known contact with patients with drug-resistant TB.

¶¶ https://www.cdc.gov/tb/topic/drtb/default.htm.
